# Physical properties of short chain aqueous organosulfate aerosol†

**DOI:** 10.1039/d3ea00088e

**Published:** 2023-08-09

**Authors:** Alison Bain, Man Nin Chan, Bryan R. Bzdek

**Affiliations:** a School of Chemistry, University of Bristol Bristol UK b.bzdek@bristol.ac.uk; b Earth System Science Programme, Faculty of Science, The Chinese University of Hong Kong Hong Kong China; c The Institute of Environment, Energy and Sustainability, The Chinese University of Hong Kong Hong Kong China

## Abstract

Organosulfates comprise up to 30% of the organic fraction of aerosol. Organosulfate aerosol physical properties, such as water activity, density, refractive index, and surface tension, are key to predicting their impact on global climate. However, current understanding of these properties is limited. Here, we measure the physical properties of aqueous solutions containing sodium methyl or ethyl sulfate and parameterise the data as a function of solute concentration. The experimental data are compared to available literature data for organosulfates, as well as salts (sodium sulfate and sodium bisulfate) and organics (short alkyl chain length alcohols and carboxylic acids) to determine if the physical properties of organosulfates can be approximated by molecules of similar functionality. With the exception of water activity, we find that organosulfates have intermediate physical properties between those of the salts and short alkyl chain organics. This work highlights the importance of measuring and developing models for the physical properties of abundant atmospheric organosulfates in order to better describe aerosol's impact on climate.

## Abstract 2

Most physical properties of short chain organosulfate aerosol cannot be approximated by salts or similar sized organics.

## Graphical abstract

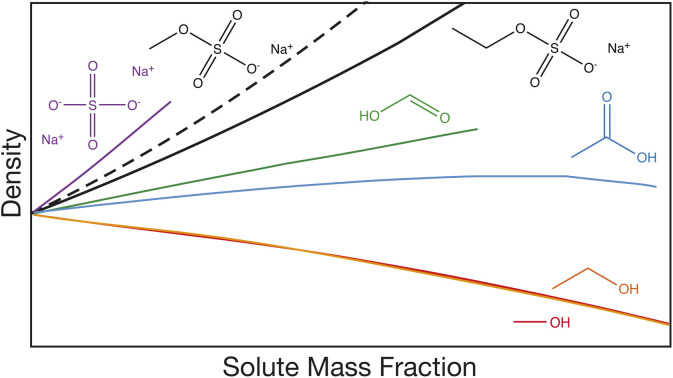

**Environmental significance**

Organosulfates are emerging as a prominent component of ambient aerosol. They can constitute up to 30% of the organic fraction of aerosol and are projected to become more important as global inorganic sulfate emissions decrease. However, little is known about how organosulfates alter the key physical properties of aerosol necessary to understand its impacts on climate. Here, we provide the first physical data and parameterisations for properties necessary to predict atmospheric impact for the two simplest organosulfates, sodium methyl and ethyl sulfates. These data and comparisons to salts and short alkyl chain organics demonstrate that the physical properties of organosulfates are intermediate between salts and organics. These results can be used to inform model development for organosulfate aerosol physical properties.

## Introduction

Aerosols are ubiquitous components of our atmosphere and are complex chemical mixtures of inorganic and organic molecules. Organosulfates are emerging as an important component of atmospheric aerosol, comprising up to 30% of the total organic mass concentration.^1^ Organosulfates have been identified in aerosol collected during field campaigns,^2–12^ and are commonly observed as key contributors to secondary organic aerosol (SOA) in laboratory studies.^1,3,13–19^ These organosulfates are formed through chemical reactions between volatile organic compounds, such as limonene, isoprene and α-pinene, with atmospheric oxidants and with sulfuric acid.^3,13–18^ Organosulfates can also be formed through reactions of fatty acids with sulfuric acid.^16^ After formation, organosulfates can be further transformed and shorter chain length alkylsulfates may be formed through fragmentation processes.^20^ Although global inorganic sulfate emissions are set to decrease, the fraction of total sulfate in the organosulfate form is predicted to increase.^3,21^ Therefore, it is important to identify the impacts of organosulfate aerosol on climate.

Despite their significance in atmospheric aerosols, systematic investigations of the physical properties of organosulfates are limited. Estillore *et al.* used a Multi-Analysis Aerosol Reactor System to measure the growth factor for a range of commercially available and synthesised organosulfate aerosol.^22^ The authors found that organosulfate aerosol does not undergo efflorescence/deliquescence behaviour (except for samples that were suspected to be contaminated with NaCl), and retains an appreciable amount of water even at relative humidities (RHs) below 10%. Ohno *et al.* investigated the hygroscopicity of supermicron organosulfate aerosol containing isoprene derived organosulfates.^23^ In agreement with the observations of Estillore *et al.*,^22^ Ohno *et al.* also found that organosulfate droplets do not undergo efflorescence nor did they observe any kinetic inhibition of water transport associated with the formation of a glassy state.^23^ This behaviour of retaining some water even at low RHs is common to highly oxidised organic molecules typically found in SOA.^24–26^ However, the observations of Ohno *et al.* show that organosulfate aerosol is more hygroscopic than SOA generated through a wide range of oxidative conditions and precursors.^23^

In atmospheric science, it is convenient to link the physical properties of aerosol to the surrounding RH. When a droplet is in equilibrium, its water activity (*a*_w_) is related to RH through eqn (1):1
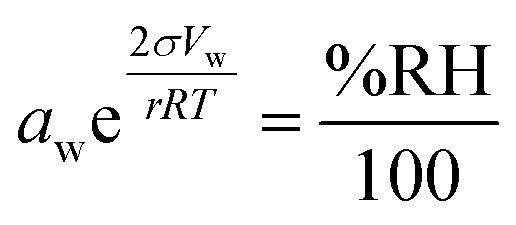
where *σ* is the surface tension, *V*_w_ is the molar volume of water, *r* is the droplet radius, *R* is the gas constant and *T* is temperature. The exponential term accounts for the Kelvin effect. When the droplet radius is larger than about 100 nm and surface curvature can be neglected, eqn (1) reduces to 
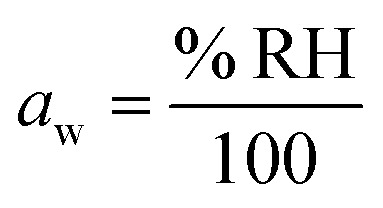
.^27^ Thermodynamic models such as Aerosol Inorganic-Organic Mixtures Functional groups Activity Coefficients (AIOMFAC) are commonly used to interchange between solute concentration and water activity.^28^ However, organosulfates currently are not a class of organic molecule functionality incorporated into AIOMFAC, likely due to a lack of experimental water activity measurements available in the literature.

Eqn (1) also shows that as aerosol experiences changing RH conditions over its atmospheric lifetime, it takes up and loses water in order to stay in equilibrium with its surroundings. The change in solute mass fraction associated with the change in water content means that its physical properties (*e.g.*, density, surface tension and refractive index) also change. These physical properties in turn affect aerosol's impact on climate.^29^ The scattering and absorption of solar radiation by aerosol (the direct effect) depend on its optical properties.^30^ Aerosol optical properties in turn depend on solute concentration and size, which are both affected by a change in RH.^31^ For aerosol which is transparent in the visible region, the real part of the refractive index is necessary to predict the direct effect of aerosol on climate.^31^ Aerosol can also indirectly impact climate by altering cloud properties. Many atmospherically relevant organic molecules reduce the surface tension of water and may be present in large enough quantities in aerosol to reduce the surface tension,^32,33^ thereby lowing the barrier to cloud droplet activation.^34–38^ A lack of experimental data for organosulfates impedes specific predictions for this class of molecules. As a result, the broad impacts of organosulfates on the aerosol direct and indirect effects are poorly understood.

Here, we measure the physical properties relevant to atmospheric aerosol (water activity, refractive index, density and surface tension) for the two simplest organosulfates: sodium methyl sulfate and sodium ethyl sulfate. These short alkyl chain organosulfates have been identified in ambient aerosol^39^ and act as proxies for the fragmentation products (due to atmospheric processing, *e.g.* oxidation) of atmospherically abundant organosulfates. Indeed, there has been much interest in investigating the reaction pathways and hygroscopic growth of these simple organosulfates under atmospheric conditions.^22,40^ Furthermore, the structural simplicity of these organosulfates allows fundamental investigation of the impact of the sulfate group on the physical properties of aqueous organics. Here, we provide parameterisations for these physical properties as a function of solute mass fraction. Mixing rules are widely used to predict the physicochemical properties of aerosols, which are complex chemical mixtures. The physical properties of binary (solute-water) solutions are generally the fundamental inputs into these mixing rules.^30,41–44^

## Experimental methods

A range of physical properties were measured for aqueous sodium methyl and ethyl sulfate solutions. We use macroscopic techniques to measure the water activity, density and refractive index of aqueous solutions. This allows high accuracy in the concentration of the solute during measurement (the uncertainty comes only from uncertainty of the masses of solution components from the analytical balance). The high accuracy in solution composition then allows high quality parameterisations of physical properties with solute concentrations. To measure surface tension, we use the droplet coalescence methods to measure the surface tension in the droplet phase.^45^ Measuring surface tension at the single droplet level allows us to determine if bulk depletion and size-dependent surface tension is important for droplets containing short alkyl chain organosulfates.

Sodium methyl sulfate (Sigma Aldrich) and sodium ethyl sulfate (Sigma Aldrich, >98% purity) were used without further purification. Stock solutions were made near the bulk saturation limit (using heat and sonication to help dissolve the solute) in deionised water. Additional concentrations were made by serial dilution of the concentrated stock solutions.

The density (*ρ*) of each solution was measured with a density meter (Densito METTLER TOLEDO) and the refractive index at 589 nm, *n*(589 nm), was measured with a refractometer (PA201, MISCO). Density and refractive index data were parameterised as a function of solute mass fraction, *w*_s_, using second order polynomials.^44^

2 *ρ* = *ρ*_0_ + *ρ*_1_*w*_s_ + *ρ*_2_*w*_s_^2^

3 *n*(589 nm) = *n*_0_ + *n*_1_*w*_s_ + *n*_2_*w*_s_^2^

The water activity, *a*_w_, of aqueous sodium methyl and ethyl sulfate solutions were measured using a benchtop activity meter (rotronic HYGROPALM23-AW). The instrument was calibrated before use with 10, 35, 50, and 80% humidity standards (aqueous LiCl and LiBr, rotronic). All measurements were collected in full equilibrium mode (equilibrium is defined as a change of <0.0005 *a*_w_ units per min) at room temperature, 295 ± 1 K.

A holographic optical tweezers instrument was used to measure droplet surface tensions using the droplet coalescence method.^37,45^ These experiments require first confining two aerosol droplets in separate optical traps generated by the use of the spatial light modulator (SLM). The phase pattern on the SLM is changed to coalesce the two droplets in a controlled manner. Upon coalescence, the cavity-enhanced Raman spectrum is collected and fit with Mie theory to determine the radius (*r*) and refractive index of the composite droplet.^46,47^ Parameterisations of refractive index are then used to determine the droplet's density (*ρ*) and solute molar concentration (*c*). Concurrently, the elastic backscattered light is collected with high time-resolution with a photodiode. The oscillatory change in shape of the composite droplet upon coalescence causes changes in the intensity of the backscattered light. The frequency (*ω*_*l*_) of the *l* = 2 surface mode oscillation relates to the surface tension (*σ*, eqn (4)).4
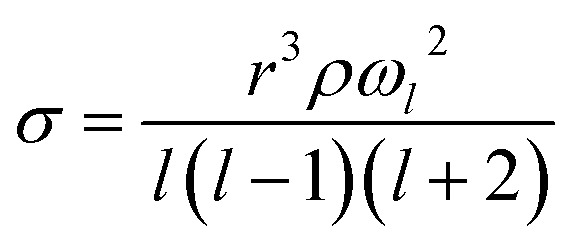


Once parameterisations for refractive index and density are known, this technique allows us to measure the surface tension using small liquid volumes. Furthermore, by measuring the surface tension in the droplet phase, we can determine whether or not bulk depletion, which becomes important as surface-area-to-volume increases for some surface active molecules,^37^ is important to consider for these solutes.

Surface tension data were fit with the Langmuir–Frumkin isotherm:^48^5
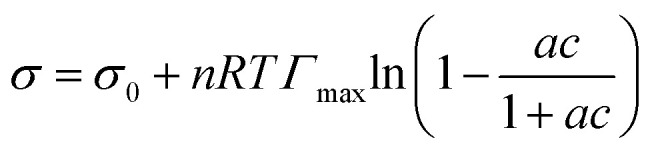
where *Γ*_max_ is the maximum surface excess (mol m^−2^), *a* = *β*/*α* (with units m^3^ mol^−1^) is the ratio of adsorption (*β*) to desorption (*α*) rate constants, *σ*_0_ is the solvent surface tension (72.8 mN m^−1^ for water), *σ* is the surface tension at the solute concentration *c* (mol m^−3^), *R* is the gas constant, *T* is the temperature (set to 298 K) and *n* = 2 for ionic surfactants.

## Results and discussion

The physical properties of relevance to organosulfate aerosol were measured for aqueous sodium methyl and ethyl sulfate solutions. Sodium methyl and ethyl sulfate represent the two simplest organosulfates and measurements of their hygroscopic growth have previously been reported by Estillore *et al.*^22^ We add to this hygroscopicity response data measurements of water activity as well as solution density, refractive index and surface tension as a function of solute concentration. Finally, using the determined water activities and densities for sodium methyl and ethyl sulfates, we calculate hygroscopic growth factors and compare our measurements with the results of Estillore *et al.*^22^

### Water activity

**Fig. 1:**
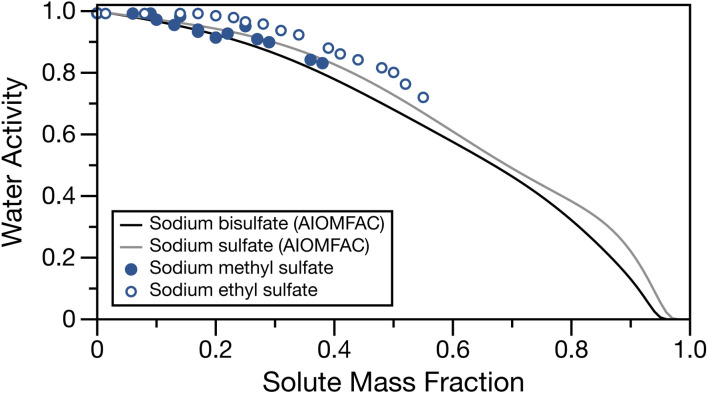
Water activity of sodium methyl sulfate (filled points) and sodium ethyl sulfate (open points) as a function of solute mass fraction. Water activity for sodium bisulfate (black line) and sodium sulfate (grey line) calculated at 295 K with AIOMFAC^49^ have been over overlayed for comparison.

The water activity of bulk sodium methyl and ethyl sulfates are tabulated in Table S1† and shown in Fig. 1 as a function of solute mass fraction. The water activities for sodium sulfate and sodium bisulfate solutions calculated at 295 K with AIOMFAC^28,49^ are also shown for comparison. These salts, having a sulfate group and associated sodium ion(s) are similar in molecular weight and structure to the low molecular mass organosulfates studied here (molecular masses of sodium bisulfate, sodium sulfate, sodium methyl sulfate and sodium ethyl sulfate are 120.06, 142.08, 134.06 and 148.11 g mol^−1^, respectively). For both organosulfates, we observe the expected behaviour of increasing water activity with decreasing solute mass fraction. For sodium methyl sulfate, even at concentrations approaching the bulk solubility limit, the water activity is only lowered to about 0.83 activity units. We note that the solubility limits of sodium methyl and ethyl sulfates are not known. The highest concentrations of solute used for these measurements are likely to be close to the solubility limit, as they required significant amounts of heat and sonication in order to completely dissolve the solute. There is close similarity between the experimentally measured water activities for sodium methyl sulfate and the AIOMFAC calculations for sodium sulfate and sodium bisulfate, and a similar shape to the activity curve is observed for sodium methyl sulfate and sodium sulfate. The measured water activities for sodium ethyl sulfate are larger than those for sodium methyl sulfate and the two inorganic sodium salts at the same solute mass fraction, likely due to the additional organic fraction and therefore reduced ion fraction at a given solute mass fraction.

Fig. S1† shows a comparison of the experimental data with soluble organics having similar alkyl chain lengths but an alcohol or carboxylic acid functional group calculated with AIOMFAC^28,49^ and sum square errors (SSE) between experimental data and the calculated water activities using AIOMFAC can be found in Table S5.† Generally, the water activities of the aqueous organics tend to overpredict the measured water activities for the organosulfates. The good agreement between measured water activities for sodium methyl and ethyl sulfate and the AIOMFAC predictions for inorganic sodium salts suggests that, until a thermodynamic model for low molecular mass organosulfates is developed, their water activities can be approximated with one of these inorganic salts. We note that for the case of sodium ethyl sulfate, many of the SSEs are low, but the shape of the water activity measurements most closely matches the sodium sulfate AIOMFAC line. We stress that this observation is in the subsaturated region and future work should characterise the water activity in supersaturated droplets.

### Density

The densities of aqueous sodium methyl and ethyl sulfate solutions were measured with a density meter and are tabulated in Tables S2 & S3.† These measurements are parameterised as a function of solute mass fraction using eqn (2) (fitting parameters in Table 1). Previous work from Cai and co-workers suggested density should be parameterised as a function of the square root of solute mass fraction.^42,52^ They found parameterising in this space to improve the accuracy of the extrapolated pure component melt density, especially for sparingly soluble solutes. The organosulfates investigated here are highly soluble and measurements of density were made at solute mass fractions up to 0.38 and 0.56 for sodium methyl and ethyl sulfates, respectively. The extrapolated pure component densities from the quadratic fit in solute mass fraction were found to agree with the extrapolated fits of cubic and quartic functions in square root solute mass fraction space.

Parameterisations of density (g cm^−3^) and refractive index at 589 nm with solute mass fraction (*w*_s_) and fit parameters for surface tension (N m^−1^) with the Langmuir isotherm^a^

**Table:** 

Density (*ρ*)	*ρ*_0_ (g cm^−3^)	*ρ*_1_ (g cm^−3^)	*ρ*_2_ (g cm^−3^)
Sodium methyl sulfate	0.9993	0.5674	0.3661
Sodium ethyl sulfate	0.9987	0.4622	0.1685

a Note *c* for the Langmuir isotherm fit is in SI units, mol m^−3^. *R*^2^ of density and refractive index fits are >0.999. Sum squared error (SSE) of Langmuir isotherm fits for surface tension are 0.00166 and 0.00097 N m^−1^ for sodium methyl and ethyl sulfate, respectively.

**Table:** 

*n*(589 nm)	*n*_0_	*n*_1_	*n*_2_
Sodium methyl sulfate	1.3331	0.0900	0.0239
Sodium ethyl sulfate	1.3330	0.0850	0.0222

**Table:** 

Surface tension	*Γ*_max_ (mol m^−2^)	*a* (m^3^ mol^−1^)
Sodium methyl sulfate	9.97 × 10^−7^	0.0027
Sodium ethyl sulfate	8.52 × 10^−7^	0.0189

**Fig. 2:**
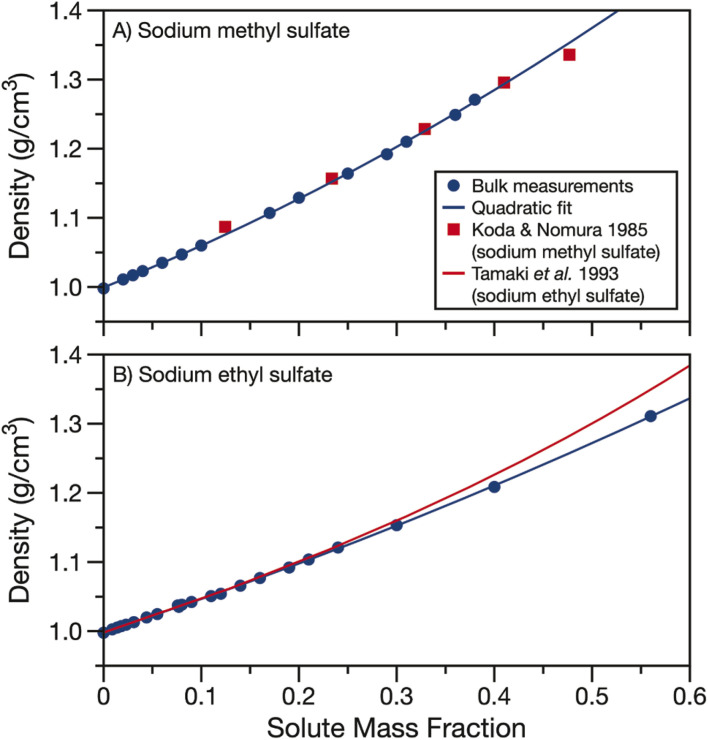
Density of aqueous (A) sodium methyl sulfate and (B) sodium ethyl sulfate. Quadratic fits of bulk data (parameters in Table 1) are shown as solid blue lines. Density data from Koda and Nomura for sodium methyl sulfate^50^ (red squares) and density calculated from the partial molar volumes given by Tamaki *et al.* for sodium ethyl sulfate^51^ (red line) are included for comparison.

The measured densities and fits are compared to data and parameterisations in the literature in Fig. 2. In the case of sodium methyl sulfate, Fig. 2A, the density measurements from Koda and Nomura^50^ agree well with the densities measured here. In the case of sodium ethyl sulfate, Fig. 2B, a parameterisation for density using the partial molar volume of sodium ethyl sulfate provided by Tamaki *et al.* is plotted to compare to the bulk measurements.^51^ This parameterisation agrees well with the measured densities when the solute mass fraction is less than about 0.3. However, as the solute concentration is increased, the partial molar volume parameterisation and experimentally determined densities begin to diverge. Using partial molar volumes to determine density leads to an over prediction compared to the measurements at high solute mass fractions.

In Fig. S2,† we compare the parameterisation of the measured densities for sodium methyl and ethyl sulfate to density data from the CRC handbook for sodium sulfate, methanol, ethanol, formic acid and acetic acid.^53^ The organic solutes were chosen for their similar alkyl chain length but different functional group replacing the sulfate. Fig. S2† clearly demonstrates the importance of determining densities for organosulfates. The densities for sodium methyl and ethyl sulfates fall in between the higher densities of sodium sulfate solutions and lower densities of solutions with other short alkyl chain length organics. This large difference in density between organosulfates and other aqueous short alkyl chain organics is at least in part due to the difference in phase state of the pure components. Sodium methyl and ethyl sulfates are solid at room temperature while the alcohols and carboxylic acids are liquids, having much lower pure component densities.

### Refractive index

**Fig. 3:**
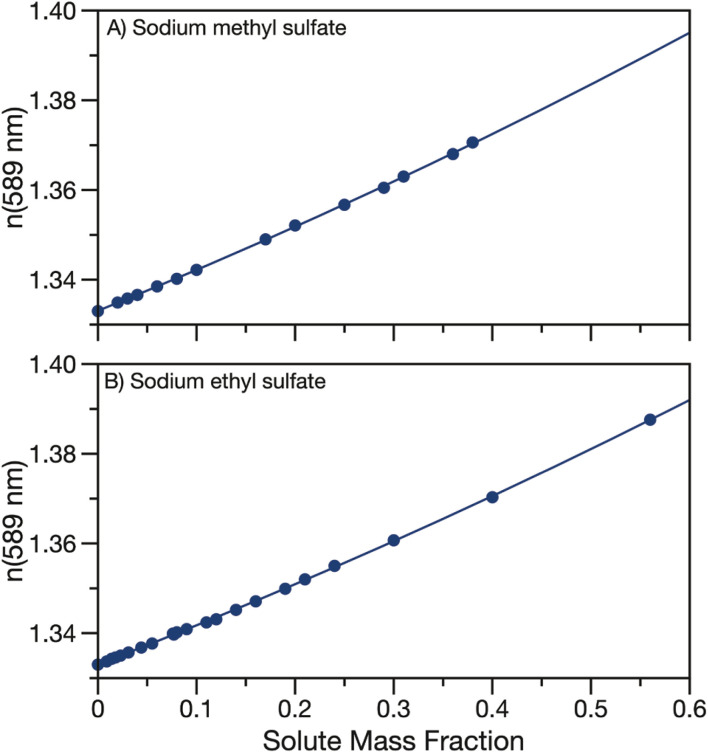
Refractive index at 589 nm for aqueous (A) sodium methyl sulfate and (B) sodium ethyl sulfate. Quadratic fits to the data (parameters in Table 1) are overlayed.

The real part of the refractive index at 589 nm was also measured for aqueous solutions of sodium methyl and ethyl sulfate. Aqueous solutions of sodium methyl and ethyl sulfate are clear and colourless which implies that the imaginary part of the refractive index is quite small and can be approximated as zero in the visible region. Data for the real part of the refractive index at 589 nm are tabulated in Tables S2 & S3† and shown in Fig. 3. As expected, as the solute concentration is increased, the refractive index also increases. Measured refractive indices are parameterised as a function of solute mass fraction using eqn (3) and the fit parameters are given in Table 1.

Fig. S3† compares the parameterisation of the measured refractive indices for sodium methyl and ethyl sulfate to data from the CRC handbook for sodium sulfate, methanol, ethanol, formic acid and acetic acid.^53^ Similarly to density, the refractive indices for the measured organosulfates fall in between sodium sulfate and the short alkyl chain length organics.

With the density and refractive index at 589 nm known, the molar refractivity, as defined in the Lorentz–Lorenz equation can be determined:^43^6
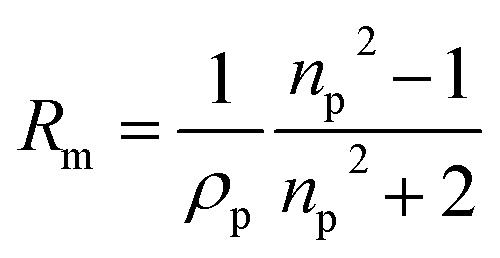
where *R*_m_ is the molar refractivity, *ρ*_p_ is the pure component density and *n*_p_ is the refractive index of the pure component. We use the density and *n*(589 nm) parameterisations in Table 1 with a solute mass fraction of one to calculate molar refractivities for sodium methyl sulfate and sodium ethyl sulfate to be 0.1403 and 0.1618 cm^3^ g^−1^, respectively. These molar refractivities can be used with the Lorentz–Lorenz molar refraction mixing rule to predict the refractive index at 589 nm for complex mixtures including additional solutes.

### Surface tension

**Fig. 4:**
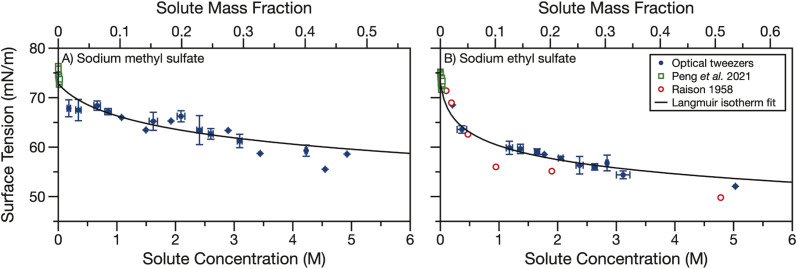
Surface tension of aqueous (A) sodium methyl sulfate and (B) sodium ethyl sulfate measured by coalescing two droplets in holographic optical tweezers (blue points). Data are binned into 0.25 M concentration bins and error bars represent the standard deviation of all datapoints in a bin. A Langmuir isotherm fit of the droplet data is shown in black. Surface tension measurements from Peng *et al.*^55^ (open green squares) and Raison^54^ (open red circles) are overlayed for comparison.

Finally, we measured the surface tension of aqueous sodium methyl and ethyl sulfate. Sodium organosulfates, like other aqueous organic molecules, are expected to have some surface activity. The magnitude of the surface activity has been previously found to depend strongly on the length of the carbon tail for linear alkylsulfates of chain length 2–18.^54^ We use holographic optical tweezers to coalesce optically trapped droplets and use the resulting elastic and inelastic light scattering to determine the surface tension. Droplet radii are in the range of 5.5–10 μm. The surface tension data shown in Fig. 4 have been averaged in 0.25 M concentration bins. The data points show the average concentration and surface tension in a bin and error bars represent the standard deviations of all datapoints in a bin. Each dataset was fit to the Langmuir–Frumkin isotherm (eqn (5)) and fit parameters are shown in Table 1.

Fig. 4 also compares our surface tension measurements for picolitre volume droplets to literature data. Peng *et al.* measured the surface tension of aqueous sodium methyl and ethyl sulfate at concentrations below 40 mM.^55^ For both sodium alkyl sulfates, they report surface tensions between 71–76 mN m^−1^ and observe no trend over the small solute concentration range examined. The solute concentrations measured by Peng *et al.* are lower than the concentrations measured here (up to 5 M) but the datasets are consistent. Our measurements suggest one would not expect to observe any change in surface tension from that of water for solute concentrations <40 mM. This observation is in reasonable agreement with the results of Peng *et al.*, which, when averaged, yield 74 ± 1 and 73 ± 1 mN m^−1^ for sodium methyl and ethyl sulfate, respectively, in good agreement with the surface tension of water, 72.8 mN m^−1^.

The surface tension of aqueous sodium ethyl sulfate was also previously measured by Raison.^54^ Surface pressure data were extracted using a digitiser from Fig. 3 in their paper and converted into surface tension using a surface tension of 72.8 mN m^−1^ for water. This dataset covers sodium ethyl sulfate concentrations of 0.1–5 M and shows some surface activity. The surface tension is reduced to about 50 mN m^−1^ after 5 M sodium ethyl sulfate is added. These data agree reasonably well with the droplet data we present here. Additionally, we find that sodium methyl sulfate is less surface active than sodium ethyl sulfate. Since the hydrophobic section of this molecule is only a methyl group, it is not surprising that it is less surface active than the ethyl sulfate. This observation is also in agreement with the trend observed by Raison for linear alkyl organosulfates with tail lengths of 2–18 carbons.^54^

Though there is some scatter in the measured surface tensions, the experimental data fit reasonably well to the Langmuir isotherm. The fit parameters in Table 1 show that the maximum surface excess, *Γ*_max_ is small (<1 × 10^−6^ m^2^ mol^−1^) compared to strong surfactants (on the order of 3 × 10^−6^ m^2^ mol^−1^*e.g.*, sodium dodecyl sulfate, SDS, a common surfactant and organosulfate^56^). This small maximum surface excess means the molecules at the surface are not tightly packed. Additionally, the ratio of kinetic parameters, *a*, is less than one, indicating the desorption rate constant is larger than the adsorption rate constant. For strong surfactants *a* can be orders of magnitude greater than one.^57,58^

In Fig. S4† we compare the Langmuir isotherm fit of the experimental surface tension data with surface tension data for short alkyl chain length organics from the CRC handbook^53^ and calculated surface tensions for sodium sulfate and bisulfate using the Extended Aerosol Inorganics Model (E-AIM).^59,60^ Like other salts, sodium sulfate and bisulfate increase the surface tension with increasing salt concentration. Conversely, short alkyl chain water soluble organics with alcohol or carboxylic acid functional groups decrease the surface tension. While the organosulfates also decrease the surface tension, the reduction in surface tension is not as strong for the same mass fraction of organosulfate as any of the other short chain organics shown in Fig. S4.† The lower surface activity of short alkyl chain organosulfates than other short alkyl chain organics can likely be explained by the large number of hydrogen bonds available with the sulfate ion, making the organosulfates more hydrophilic than other short alkyl chain organics. Indeed, sulfate ions are known to preferentially reside in the bulk of aqueous solutions.^61^

In high surface-area-to-volume ratio droplets, surfactants can become depleted in the droplet bulk as a large proportion of the total surfactant concentration partitions to the interface.^37,62^ The agreement between our droplet measurements and bulk measurements from Peng *et al.*^55^ and Raison,^54^ in addition to the weak surface activity we observe, indicates that bulk depletion is not significant for sodium methyl or ethyl sulfate in 5.5–10 μm radius droplets. We further investigated the potential effects of bulk depletion using a framework described by Alvarez *et al.*^63^ The bulk depletion ratio is defined as the ratio of surfactant concentration in a droplet's bulk to the concentration in the bulk of a macroscopic solution when the total concentration (bulk + surface concentrations) is the same. In this framework, bulk depletion ratios are >0.99 for 5 μm radius droplets containing 50 mM total sodium methyl and ethyl sulfate concentration, indicating that this droplet can be treated as a macroscopic solution and the surface concentration can be neglected. Depletion becomes even less significant as surfactant concentration and/or droplet radius increase.^62,63^ Since no depletion is expected when the concentration is 50 mM, depletion will not occur in the measured droplets where the organosulfate concentration is much higher. Behaving similarly to weakly surface active organics, we could still expect depletion to occur in smaller droplets.^64^ The surface tension measurements presented here could be used in the future to predict the surface-bulk partitioning of organosulfates in smaller droplet sizes and the overall impact of organosulfates on the aerosol indirect effect.

### Growth factor

Using the determined density parameterisations, the growth factor as a function of water activity can be calculated at each water activity in Table S1.† These growth factors are compared to the fits of two hygroscopic growth expressions (see Varutbangkul *et al.*^65^ and Kreindenweis *et al.*^66^) of the growth factor data performed by Estillore *et al.* for their hygroscopic growth data.^22^ Growth factor was calculated by assuming a 100 nm dry diameter (*D*_0_) of sodium methyl or ethyl sulfate having densities of 1.932 and 1.629 g cm^−3^ (setting solute mass fraction = 1 in the density parameterisations), respectively. These values may underestimate the solid density, but the solid densities for these molecules were not available from the manufacturer. The solute was assumed non-volatile, and the solute and water mass fractions for each water activity measurement were used with the solution density to calculate the wet particle diameter (*D*_p_). Calculated growth factors are tabulated in Table S1.†

**Fig. 5:**
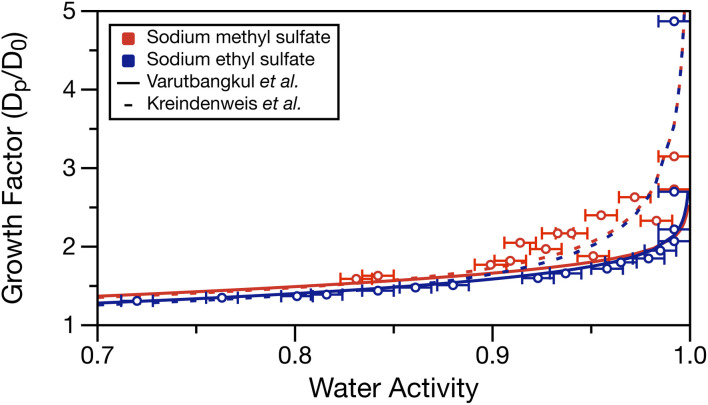
Calculated growth factor (ratio of wet, *D*_p_, and dry, *D*_0_, diameters) for measured water activities in Table S1.† Error bars in the *x*-direction represent the measurement uncertainty on water activity. Dashed and solid lines show the growth factor parameterisations from the work of Estillore *et al.*^22^ using the equations of Varutbangkul *et al.*^65^ and Kreindenweis *et al.*^66^

Fig. 5 overlays the growth factor fits by Estillore *et al.* of their growth factor data^22^ and the calculated growth factors from the water activity data in Table S1.† The growth factor data from Estillore *et al.* was limited to RHs below 90%, which accounts for the divergence of the two growth factor parametrisations as the RH approaches 100%. In the case of sodium methyl sulfate, there is some spread in the data points caused by the noise in the water activity measurements. Nonetheless, a clear trend of increasing growth factor with increased water activity exists, as expected. The calculated growth factors for sodium methyl sulfate appear to be in better agreement with the growth factor parametrisation of Kreindenweis *et al.*^66^ In the case of sodium ethyl sulfate, the expected trend of increasing growth factor with increased water activity is again observed. Here, excellent agreement exists between the calculated growth factors and the parameterisation of Varutbangkul *et al.*^65^ This comparison corroborates the growth factor observations of Estillore *et al.*^22^ and provides further validation of the density and water activity measurements presented here.

## Conclusion

Organosulfates are emerging as an important component of atmospheric aerosol. It is necessary to understand their chemical and physical properties to estimate their impact on global climate. In this study, we investigated the physical properties of the two simplest sodium salt organosulfates, sodium methyl and ethyl sulfate, as a function of solute concentration. Measured water activities of aqueous sodium methyl and ethyl sulfate solutions were quite similar to the water activities of sodium sulfate and sodium bisulfate, suggesting the water activities of these salts can be used to approximate the water activity of low molecular weight organosulfates until activity parameterisations become available. Aqueous solution densities for sodium methyl sulfate were in close agreement with measurements from Koda and Nomura,^50^ but the predicted density of sodium ethyl sulfate using partial molar volumes from Tamaki *et al.*^51^ diverged from the measured densities when the solute mass fraction was above 0.3. The refractive indices at 589 nm for sodium methyl and ethyl sulfate were similar to one another. Molar refractivities were determined to be 0.1403 and 0.1618 cm^3^ g^−1^ for sodium methyl sulfate and sodium ethyl sulfate, respectively. These molar refractivities can be used with the Lorentz–Lorenz molar refraction mixing rule to predict the refractive index of aerosol containing organosulfates in addition to other solutes. Finally, through measurement of droplet surface tension, both organosulfates are shown to be weakly surface active. This observation is in agreement with available literature data and matches the previously observed trend of increasing surface activity with increasing carbon tail length for linear sodium alkylsulfates with C2–C18 tails.^54^

By comparing the physical property data measured here for sodium methyl and ethyl sulfates to literature data for aqueous sodium sulfate and bisulfate as well as aqueous organics with similar alkyl chain lengths but alcohol or carboxylic acid functional groups, we see that organosulfates have intermediate physical properties between inorganic sulfate salts and short alkyl chain organics. With the exception of water activity, using the physical properties data from sodium sulfate or bisulfate would overestimate the physical properties of these organosulfates, whereas using the physical properties data of other short chain organics would underestimate their physical properties. This observation highlights the importance of determining the physical properties of atmospherically abundant organosulfates. Organosulfate functionality is not currently included in group contribution models for aerosol physical properties. However, these results show that this is a necessary addition to current frameworks, as most physical properties cannot be approximated either by sulfate salts or similarly sized organics. Together, these data provide a reference point for the physical properties of organosulfates in atmospheric aerosol and can be used to approximate the physical properties of organosulfates in ambient aerosol in order to make predictions of their climate impacts through their incorporation in mixing rule calculations of physical properties. Future work will investigate the physical properties of higher molecular weight organosulfates and target the supersaturated concentrations under which ambient aerosol can exist.

## Conflicts of interest

There are no conflicts to declare.

## Supplementary Material

EA-003-D3EA00088E-s001
