# Supplementary material for: Physical properties of short chain aqueous organosulfate aerosol
Source: Environ Sci Atmos. 2023 Aug 9;3(9):1365–73. doi: 10.1039/d3ea00088e (PMC10500313; doi:10.1039/d3ea00088e)
Supplement: EA-003-D3EA00088E-s001 [file EA-003-D3EA00088E-s001.pdf]

Electronic Supplementary Material (ESI) for Environmental Science: Atmospheres.
This journal is © The Royal Society of Chemistry 2023

Supplementary Information

"Physical Properties of Short Chain Aqueous Organosulfate Aerosol"

Environmental Science: Atmospheres

Alison Bain,[1] Man Nin Chan,[2,3] and Bryan R. Bzdek[1]

[1] School of Chemistry, University of Bristol, Bristol, United Kingdom
[2] Earth System Science Programme, Faculty of Science, The Chinese University of Hong Kong, Hong Kong, China
[3] The Institute of Environment, Energy and Sustainability, The Chinese University of Hong Kong, Hong Kong, China

Table S1: Measured water activity (±0.008 a_w units) for sodium methyl and ethyl sulfates at various solute mass fractions and calculated growth factors.

Sodium methyl sulfate			Sodium ethyl sulfate		
Solute mass fraction	Water activity	Growth factor (D_p/D_0)	Solute ass fraction	Water activity	Growth factor (D_p/D_0)
0	0.993		0	0.993	
0.060	0.992	3.15	0.014	0.992	4.87
0.090	0.992	2.73	0.080	0.992	2.70
0.10	0.972	2.63	0.14	0.992	2.22
0.13	0.955	2.40	0.17	0.992	2.07
0.14	0.983	2.33	0.20	0.985	1.95
0.17	0.940	2.17	0.23	0.979	1.85
0.17	0.933	2.17	0.25	0.965	1.80
0.20	0.914	2.07	0.28	0.958	1.72
0.22	0.927	1.97	0.31	0.937	1.66
0.25	0.951	1.88	0.34	0.923	1.60
0.27	0.909	1.82	0.39	0.880	1.51
0.29	0.899	1.77	0.41	0.861	1.48
0.36	0.842	1.63	0.44	0.842	1.44
0.38	0.831	1.59	0.48	0.816	1.39
			0.50	0.801	1.37
			0.52	0.763	1.35
			0.55	0.720	1.31

*Table S2: Physical parameters for sodium methyl sulfate measured with bulk techniques. *Error on density measurements is 0.0005 g/cm³ **Error on n(589nm) measurements is 0.00005 RI units.*

Solute Mass Fraction	Molar Concentration (M)	Density* (g/cm^3)	n(589nm)**
0.00	0.00	0.998	1.3330
0.02	0.15	1.011	1.3349
0.03	0.23	1.017	1.3358
0.04	0.30	1.023	1.3366
0.06	0.45	1.035	1.3385
0.08	0.61	1.047	1.3402
0.10	0.79	1.060	1.3422
0.17	1.40	1.107	1.3490
0.20	1.68	1.129	1.3521
0.25	2.17	1.164	1.3567
0.29	2.58	1.192	1.3605
0.31	2.80	1.210	1.3630
0.36	3.35	1.249	1.3680
0.38	3.60	1.271	1.3706

*Table S3: Physical parameters for sodium ethyl sulfate measured with bulk techniques. *Error on density measurements is 0.0005 g/cm^3 **Error on n(589nm) measurements is 0.00005 RI units.*

Solute Mass Fraction	Molar Concentration (M)	Density* (g/cm^3)	n(589nm)**
0.00	0.00	0.998	1.3330
0.0087	0.071	1.003	1.3337
0.014	0.11	1.005	1.3343
0.018	0.14	1.007	1.3346
0.023	0.18	1.009	1.3350
0.031	0.25	1.013	1.3357
0.044	0.36	1.020	1.3368
0.055	0.45	1.025	1.3377
0.076	0.63	1.037	1.3399
0.077	0.63	1.035	1.3397
0.08	0.66	1.038	1.3402
0.09	0.74	1.043	1.3409
0.11	0.92	1.051	1.3424
0.12	1.00	1.054	1.3630
0.14	1.18	1.066	1.3452
0.16	1.37	1.077	1.3471
0.19	1.65	1.092	1.3499
0.21	1.84	1.104	1.3520
0.24	2.13	1.121	1.3550
0.30	2.74	1.153	1.3607
0.40	3.83	1.209	1.3703
0.56	5.82	1.311	1.3876

Table S4: Surface tensions of 5.5 – 10 µm radius aqueous sodium organosulfates droplets measured with holographic optical tweezers. Tabulated concentrations and surface tensions are averages and standard deviations for 0.25 M bins. Where no standard devations are given, only a single point fell within the concentration bin.

Sodium methyl sulfate		Sodium ethyl sulfate	
Concentration (M)	Surface tension (mN/m)	Concentration (M)	Surface tension (mN/m)
0.18±0.03	67.9±1.7	0.21	68.5
0.34±0.05	67.5±2.2	0.37±0.08	63.6±0.8
0.66±0.06	68.4±0.9	1.18±0.05	59.9±1.3
0.85±0.06	67.2±0.6	1.36±0.05	59.6±0.9
1.08	66.0	1.65±0.04	59.1±0.6
1.49	63.5	1.77	58.5
1.62±0.07	65.2±1.8	2.05±0.04	57.8±0.3
1.92	65.3	2.37±0.06	56.3±1.8
2.09±0.07	66.2±1.1	2.63±0.04	56.0±0.6
2.41±0.05	63.4±2.9	2.84±0.02	56.8±1.6
2.60±0.04	62.7±1.1	3.12±0.11	54.4±0.8
2.90	63.4	5.03	52.1
3.09±0.04	61.3±1.4		
3.44	58.7		
4.23±0.02	59.3±1.1		
4.55	55.5		
4.92	58.6		

Table S5: Sum square errors (a_w units) between measured water activities of sodium methyl and ethyl sulfate solutions and AIOMFAC calculations for aqueous solutions of other short alkyl chain organics.

AIOMFAC calculation me	Sodium thyl sulfate	Sodium ethyl sulfate
Ethanol	0.018	0.036
Formic acid	0.021	0.057
Acetic acid	0.014	0.032
Propanoic acid	0.027	0.043
$NaHSO_4$	0.019	0.072
$(Na)_2SO_4$	0.013	0.042

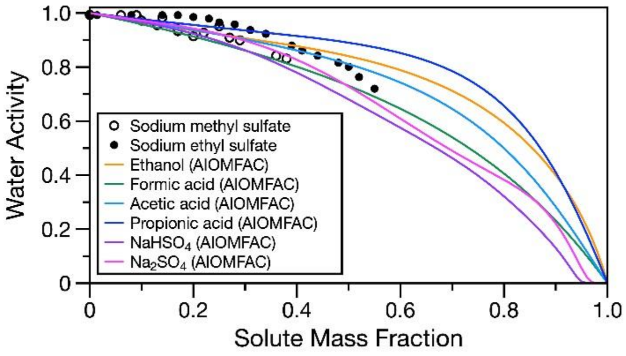

Figure S1: Comparing measured water activities for sodium methyl and ethyl sulfates to predicted water activities from AIOMFAC[1,2] for sodium sulfate and bisulfate as well as organic molecules having similar alkyl chain lengths but with an alcohol or carboxylic acid functionality.

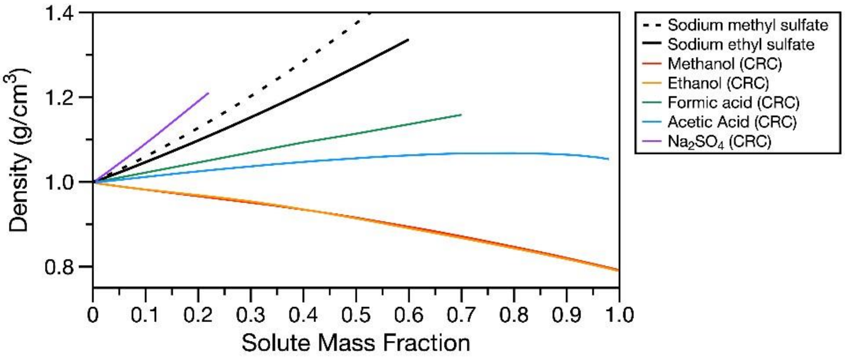

Figure S2: Comparing density parametrizations for sodium methyl and ethyl sulfates to densities from the CRC handbook[3] for sodium sulfate and organic molecules having similar alkyl chain lengths but with alcohol or carboxylic acid functionality.

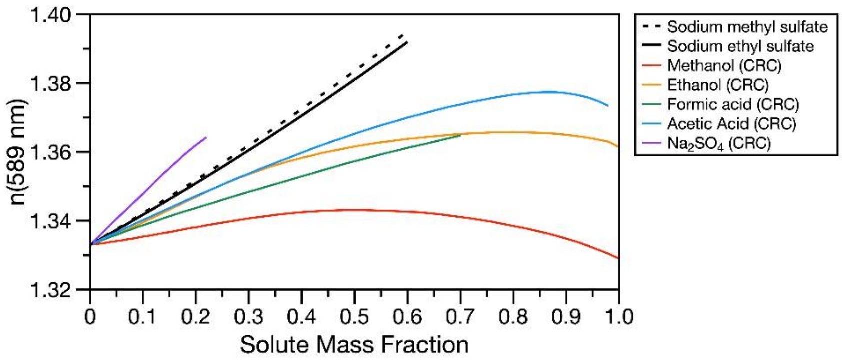

Figure S3: Comparing refractive index at 589 nm parametrizations for sodium methyl and ethyl sulfates to refractive indices from the CRC handbook for sodium sulfate and organic molecules having similar alkyl chain lengths but with alcohol or carboxylic acid functionality.

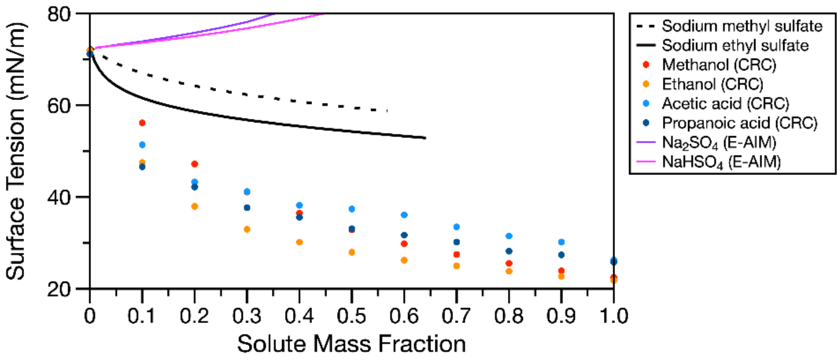

Figure S4: Langmuir isotherm fits of surface tension measurements for sodium methyl and ethyl sulfates compared to predictions for sodium sulfate and bisulfate from E-AIM [4,5] *and short alkyl chain length organics having an alcohol or carboxylic acid functional group from the CRC handbook.*[3]

References

1 A. Zuend, C. Marcolli, B. P. Luo and T. Peter, *Atmos. Chem. Phys.*, 2008, 4559–4593.

2 https://aiomfac.lab.mcgill.ca/model.html, AIOMFAC online model.

3 J. R. Rumble, Ed., *Handbook of Chemistry and Physics*, CRC Press/Taylor & Francis, Boca Raton, FL., 102nd edn., 2021.

4 C. S. Dutcher, A. S. Wexler and S. L. Clegg, *J. Phys. Chem. A*, 2010, **114**, 12216–12230.

5 Http:/www.aim.env.uea.ac.uk/aim/surftens/surftens.php, Extended AIM thermodynamic model: Surface tension of aqueous solutions.
